# A Human Biomonitoring Study Assessing Glyphosate and Aminomethylphosphonic Acid (AMPA) Exposures among Farm and Non-Farm Families

**DOI:** 10.3390/toxics10110690

**Published:** 2022-11-15

**Authors:** Alison Connolly, Holger M. Koch, Daniel Bury, Stephan Koslitz, Marike Kolossa-Gehring, André Conrad, Aline Murawski, James A. McGrath, Michelle Leahy, Thomas Brüning, Marie A. Coggins

**Affiliations:** 1 Centre for Climate and Air Pollution Studies, Physics, School of Natural Sciences and the Ryan Institute, University of Galway, University Road, H91 CF50 Galway, Ireland; 2 Institute for Prevention and Occupational Medicine of the German Social Accident Insurance, Institute of the Ruhr-University Bochum (IPA), Bürkle-de-la-Camp-Platz 1, 44789 Bochum, Germany; 3 German Environment Agency (Umweltbundesamt), 06844 Dessau-Roßlau, Germany

**Keywords:** biological monitoring, environmental exposure, exposure assessment

## Abstract

Glyphosate-based pesticides are the highest-volume used herbicides worldwide. International concerns regarding the potential human adverse effects of glyphosate exposures have heightened since IARC classified glyphosate as probably carcinogenic to humans. Human biomonitoring (HBM) studies have identified ubiquitous exposure to glyphosate and its main breakdown product, aminomethylphosphonic acid (AMPA), from environmental exposures. The IMAGE research project aimed to investigate farm and non-farm families’ exposure to glyphosate while aligning with the Human Biomonitoring for Europe (HBM4EU) initiative. The study recruited non-farm and farm families (who use glyphosate on their farms). Each family member provided a urine sample that was analysed using gas chromatography coupled with tandem mass spectrometry, with a limit of quantification of 0.05 µg/L for glyphosate and AMPA. In addition to general information on background exposures in farm and non-farm families, we investigated relationships in exposure between families and family members. We recruited 68 families, including 54 non-farm and 14 farm families (180 vs. 45 individuals). Some pesticide users (*n* = 14, all male farmers) had slightly elevated AMPA levels compared to other adult participants but, overall, we observed no significant differences between farm and non-farm families. The main metabolite, AMPA, was quantifiable in twice as many samples as glyphosate (61% vs. 32%), with a maximum concentration of 7.24 µg/L vs. 3.21 µg/L. Compared to previous studies, exposure levels were relatively low and far below current health-based guidance values (3% or less for glyphosate and AMPA). Study results suggest potential exposures from residential co-exposures or living with a pesticide user. This is the first study internationally to investigate glyphosate and AMPA across family members (farm and non-farm). We found comparably low glyphosate and AMPA exposures among these families. These results enhance our understanding of glyphosate exposures for different demographic groups and contribute to the scientific knowledge on exposures required for regulatory risk assessments and the re-evaluation of glyphosate in 2022 by the European Commission.

## 1. Introduction

Glyphosate (N-(phosphonomethyl) glycine) is a broad-spectrum herbicide and its main environmental biodegradation product is aminomethylphosphonic acid (AMPA). Since the 1970s, glyphosate-based pesticide products have been marketed and are now the highest-volume used herbicide sold worldwide [1,2,3,4]. Glyphosate is widely used in agricultural and horticultural settings, including crops, grasslands and parks, as well as for amateur and home garden use.

In 2015, the International Agency for Research on Cancer (IARC) classified glyphosate as ‘Group 2A—probably carcinogenic to humans’, significantly increasing debate on its safety [5]. However, in 2015, the European Food Safety Authority (EFSA) and the European Chemical Agency (ECHA) differed in their classification [6,7] and ECHA has since re-evaluated and confirmed that glyphosate does not meet the criteria to be classified as a carcinogen [8]. EFSA will carry out its risk assessment of glyphosate, which is scheduled to be finalised by July 2023 [9].

Scientific publications have also had some ambiguities regarding the possible adverse health effects, with some review studies concluding that exposure to glyphosate is associated with cancer in humans [10,11], while an Agricultural Health Study (AHS) survey found no statistically significant associations between glyphosate use and cancer [12]. However, studies have suggested links between glyphosate exposure and potential adverse health effects on the endocrine system [13], renal system [14,15], respiratory system [16,17] and reproductive system [18,19].

Along with the conflicting studies on the hazardous properties of glyphosate, there is also a wide variance in reported exposure levels. A recent review identified ubiquitous exposures to glyphosate and highlighted variations that are likely due to methodological differences (e.g., differing analytical techniques) and differing exposure scenarios (e.g., occupational and environmental settings) [20]. Regardless, there is an urgent need for additional exposure studies to evaluate glyphosate levels and related biomarkers in the general population and occupational groups [21].

Human biomonitoring (HBM) is the measurement of chemicals or their metabolites in biological media, such as blood, urine, hair or breast milk [22,23], and is considered an essential tool for comprehensive exposure assessment and risk management [24]. HBM data are particularly beneficial as they provide internal concentrations that can be extrapolated to total external exposures, providing reliable exposure information that can be linked to toxicological data for risk characterisation [25,26], thus, playing a pivotal role in exposure and risk management and providing essential information for regulatory agencies and policy-makers [25]. Glyphosate and AMPA have been identified as priority substances by the European Human Biomonitoring (HBM4EU) initiative [27,28], a collaboration between institutes from 30 countries, the European Environment Agency and the European Commission, co-funded under Horizon 2020 (www.hbm4eu.eu). In addition, glyphosate has also been included in national biomonitoring programmes in Canada [29], Germany [30,31] and the United States of America (USA) [32].

There are still very few HBM studies investigating glyphosate worldwide [20]. Occupational studies have identified exposures among professional pesticide users [33,34,35,36] and environmental exposures among differing adult populations [30,37,38,39]. However, fewer studies have included AMPA in their study remit [30,37,39,40,41,42], though AMPA has a similar toxicological profile as glyphosate. Studies have also reported similar urinary AMPA and glyphosate concentration levels and correlated these concentrations [30,37,40].

There is also a paucity of data regarding glyphosate and AMPA exposures among potentially more vulnerable groups, such as children, and sub-populations, such as farm families. Few studies have investigated mothers’ and children’s exposure to glyphosate [31,33,43,44]. These studies indicate exposure differences among different family members, which may be due to physiology, behaviour (e.g., home use of pesticides) and dietary factors.

For example, a recent study among German children and adolescents analysed 2144 first-morning void urine samples and approximately half were quantifiable for glyphosate and AMPA, with maximum concentrations of 1.11 µg/L and 13.4 µg/L for glyphosate and AMPA, respectively [31]. In addition, a few studies have investigated mothers’ and children’s exposure to glyphosate [31,33,43,44].

One study identified glyphosate exposures among family members in farm and non-farm families [45], with a high frequency of samples found above the limit of detection (66–88%). However, this study did not include AMPA and was in Iowa, United States, which has differing regulations and glyphosate use patterns compared to the European context. Some families might have higher levels of exposure due to living with an occupational user (e.g., farmer), as a result of residential exposure (e.g., living on a farm) or take-home (e.g., residues brought into the house via clothes/equipment) exposures. In particular, farm families might experience additional glyphosate exposures due to family members working with glyphosate or its use on the farm in close proximity to the home, as exposures can be elevated during spraying seasons [46]. This is especially important for vulnerable populations, such as children, who generally tend to exhibit higher levels of glyphosate body burdens than their adult counterparts [44,45].

This study aimed to fill this gap for assessing glyphosate and AMPA exposures among vulnerable groups (children) and within a potentially additionally exposed subgroup (farm family). This study investigated glyphosate and AMPA exposures among farm and non-farm families in Ireland. Only farms where glyphosate had been sprayed the day before were included. To the best of the authors’ knowledge, this is the first study to investigate families’ exposure to glyphosate and AMPA among Irish farm and non-farm families.

## 2. Materials and Methods

### 2.1. Study Population and Sampling Protocols

The study recruited families in Ireland that included both parents (or guardians) and at least one of their children (3–19 years of age) within each family, from both farm and non-farm families. An inclusion criterion for the farm family was that at least one family member had to spray glyphosate-based pesticide products as part of their duties on the farm the day before urine sample collection.

The recruitment campaign was advertised via a designated website (www.nuigalway.ie/image, accessed on 1 March 2022), social media and a press release to national papers and journals. Interested potential candidates were sent an invitation letter and a project information sheet to inform them about the study. Once candidates agreed to participate, a consent form was obtained from both parents, who both signed a form for themselves and on behalf of the child participant. In addition, a child assent form was developed to explain the study to the younger children within the study.

Both parents were asked to complete the IMAGE project questionnaire that included information on sociodemographic factors, environment and home exposures, dietary habits, lifestyles, occupations and health. In addition, the parents/guardians were asked to complete or assist with completing an abbreviated questionnaire for the children. A portion of the food-serving size guidance document was provided to assist with completing the dietary habits within the questionnaire.

The questionnaires, consent forms and guidance documentation were adapted from protocols developed by experts in the scientific field and peer-reviewed under HBM4EU (www.hbm4eu.eu, accessed on 1 September 2022), a Horizon-2020-funded research project [47,48]. The HBM4EU questionnaire for pesticides [49] was used, with some questions that did not apply to the current study being removed. This questionnaire was also used for children but was shortened substantially to include only the necessary questions required for the study.

### 2.2. Urine Sample Collection

For urine collection, participants were given instructions on providing a urine sample, a 500 mL plastic container and a Whirl-Pak^®^ sample bag to place the container within the bag. Each family member was asked to provide one first-morning, full-void urine sample. The farm family was asked to provide this sample the morning after one family member sprayed glyphosate-based pesticide products. Further, the pesticide-using family member provided a urine sample. Alongside the urine samples, the participants were asked to complete a contextual information sheet in relation to each urine sample, which included information on the time of the void, time of the previous void, whether the sample was complete, storage, potential activities that resulted in pesticide exposure and food types that were consumed 48 h before sample collection. Urine samples were collected from the participants’ homes.

The sampling protocols had to be amended as the COVID-19 pandemic travel restrictions commenced in Ireland shortly after the sampling campaign started. As a result, all project sampling protocols were adapted to comply with Ireland’s relevant Public Health Guidelines (https://www.gov.ie/en/publication/6973bc-daily-briefing-on-the-governments-response-to-covid-19-monday-30-mar/, accessed on 16 June 2022). Further details on the sampling protocols are described in Supplementary Information.

Project ethical approval was received from the National University of Ireland Galway Research Ethics Committee (Ref: 19-Jun-05—IMAGE—Ireland’s bioMonitoring Assessment of Glyphosate Exposure: an environmental assessment glyphosate exposure among the Irish population using biomonitoring).

### 2.3. Urine Sample Chemical Analysis

All samples were prepared and analysed for glyphosate and AMPA following the analytical method described previously [50]. In brief, urine samples (frozen at −18 °C) were thawed and homogenised directly before analysis. All solutions, including standard and internal standard solutions, were prepared and stored in polypropylene containers. After the addition of the internal standards (AMPA-^13^C,^15^N and glyphosate-_d2_) and a surplus of acetonitrile, the samples were evaporated to dryness and the residue was dissolved in and derivatised with trifluoroethanol and trifluoroacetic anhydride. Analysis was performed using gas chromatography coupled to tandem mass spectrometry (GC–MS/MS) with quantification via isotope dilution. The limit of quantification (LOQ) was 0.05 µg/L for both glyphosate and AMPA. Inter- and intra-day imprecision (coefficient of variation) was below 11% for glyphosate and below 8% for AMPA. The method’s accuracy (relative recoveries of spiked concentrations in urine, *n* = 8, two spiking levels) was between 83% and 124%.

This method was successful in its application to the German External Quality Assessment Scheme for analyses in Biological Materials (G-EQUAS) (http://www.g-equas.de, accessed on 23 February 2022) for glyphosate (does not include AMPA) and received certification of successful participation for glyphosate for the whole duration of this study (G-EQUAS rounds 64 and 65). Additionally, the method successfully participated in all three rounds of the HBM4EU (http://www.hbm4eu.eu, accessed on 1 September 2022) interlaboratory comparison investigations of selected pesticide biomarkers in human urine for both glyphosate and AMPA.

Urinary creatinine concentrations were determined by the Jaffé method (L.u.P GmbH Labor und Praxis Service, Bochum, Germany).

### 2.4. Statistical and Data Analysis

Summary and descriptive statistics were calculated on the demographic and exposure variables. For the summary statistical analysis, glyphosate and AMPA concentrations below the LOQ were not imputed because of the low detection rates. For graphical representation, the LOQ/2 was imputed. All further data analyses were conducted on dichotomous information (i.e., detects/non-detects) or using non-parametric statistical tests. Urinary concentrations of glyphosate and AMPA are summarised and presented by statistical characteristics (sample size (N), number of samples below LOQ (N < LOQ), sample fraction at or above LOQ (% ≥ LOQ), median, the 95th percentile and maximum value (Max) for both the farm and non-farm families, with subgroups by participant type (i.e., father, mother, and child)). Detection frequencies of different groups (e.g., family members or farm and non-farm families) were compared using a Chi-squared test of independence for <LOQ vs. ≥LOQ (i.e., detects vs. non-detects) of glyphosate and AMPA. Differences in frequency were considered statistically significant if *p* ≤ 0.05. Glyphosate and AMPA concentrations are presented in µg/L and urinary concentrations were adjusted for creatinine (Supplementary Materials, Table S1) and for combined families (farm and non-farm) for each family member (Supplementary Materials, Table S2). The remainder of the study results was evaluated using unadjusted urinary values (µg/L). Though creatinine is widely used to account for variations in urinary analyte concentrations from changing water content in urine, creatinine adjustments can cause a wide fluctuation due to a number of factors (e.g., sex/age/exercise) and this adjustment factor does not always ensure more accuracy for certain chemicals [51,52,53].

To evaluate urinary concentrations of glyphosate and AMPA in terms of exposure risk, the maximum concentrations found among adults and children for glyphosate and AMPA were compared to current health-based guidance values for environmental exposures, the European Food Safety Authority (EFSA) current Acceptable Daily Intake (ADI) allowance of 0.5 mg/kg bw/day [1]. To extrapolate urinary concentrations for external exposures, expressed as the mass of glyphosate (or AMPA) taken up, per kilogram of body weight per day (µg/kg bw/day), the same back-calculation approach used in Connolly, Coggins [20] was applied. The concentration of glyphosate/AMPA in urine is multiplied by the daily volume of urine (assumed as 2 L per day for adults [54] and 1.3 L for children [55]) and divided by body weight (assumed as 60 kg for adults), multiplied by the urinary excretion fraction (F_ue_) and by EFSA’s ADI (0.5 µg/kg bw/day) (Equation (1)). The child’s actual reported weight was used for the children’s value. The ADI is expressed as the mass of glyphosate taken up, per kilogram of body weight per day (µg/kg bw/day).

(1) $$\%\;\mathrm{ADI}=\frac{\mathrm{Gly}/{\mathrm{ampa}}_{\mathrm{conc}.}\times {\;\mathrm{Vol}}_{\mathrm{Urine}}}{\mathrm{BW}\times F_{\mathrm{ue}}\times \mathrm{ADI}}$$

where Gly/ampa_conc_ is the concentration of glyphosate or AMPA measured in urine; Vol_urine_ is assumed as two litres for adults and 1.3 L for children [55]; BW is bodyweight which is standardised at 60 kg; the F_ue_ is the urinary excretion fraction (set for glyphosate to 1% and AMPA to 23% [56]). ADI is the acceptable daily intake allowance (for glyphosate, it is 0.5 µg/kg bw/day).

## 3. Results

### 3.1. Descriptive and Summary Statistics

The study had a total of 68 families throughout the Republic of Ireland participate, including 54 non-farm families and 14 farm families (with glyphosate having been sprayed the day before sampling), a total of 226 individuals altogether (180 non-farm, 46 farms), collected from 2019 to 2020. Two mothers signed consent forms but opted out of the study before providing a urine sample (one non-farm and one farm family), reducing the total number of individuals included in this study to 224. Each family had at least one child participating, but the study accepted samples from multiple children within a family if consented to by the parents/guardians. Additionally, one child from a non-farm family gave two samples (as they initially missed the first-morning void). In total, 92 children and adolescents participated in the study, 74 non-farm (with one child giving two urine samples on two different days) and 18 farm families. Each participant completed a questionnaire, including personal information (e.g., age/weight/height), dietary habits, personal use of pesticides and health questions. The age range of the children and adolescents within this study was from 3 to 19 years old (Table 1). Among the 14 participating farm families, it was exclusively the male that had been using glyphosate products the day before sampling. A total of 227 urine samples was collected, 93 samples from the children and the remainder from the adult groups.

### 3.2. Glyphosate and AMPA Urinary Concentrations

All urinary glyphosate and AMPA concentrations are provided in Table 2 in unadjusted concentrations (µg/L), presented as non-farm and farm families, sub-grouped by participant type (i.e., father, mother and child). For glyphosate, the percentage above the limit of quantification (LOQ = 0.05 µg/L) ranged from 17% to 36% and 17% to 43% among non-farm and farm families, respectively. The maximum value detected for glyphosate was 3.21 µg/L, which was for a farm father who was spraying glyphosate-based pesticide products (i.e., the five-highest glyphosate concentrations were from this group). For AMPA, there was a higher percentage of samples above the LOQ compared to glyphosate, ranging from 59% to 60% and 38% to 67% among non-farm and farm families, respectively. The maximum reported value for AMPA was 7.24 µg/L, which was for a non-farm child. Concentrations after creatinine adjustment are provided in the Supplementary Information.

**Figure 1:**
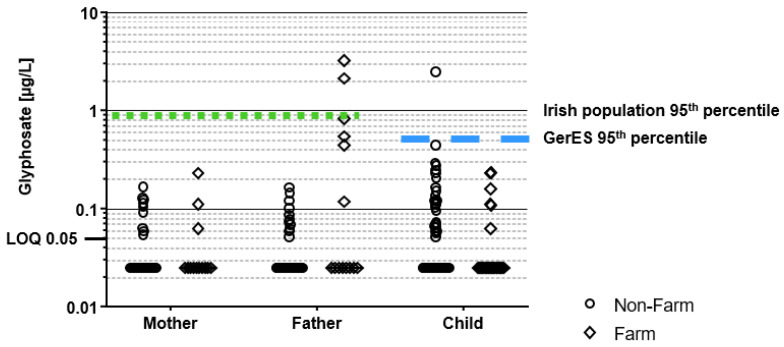
Urinary concentrations of glyphosate by the family member (e.g., mother, father, child) and family type (e.g., farm and non-farm). Comparisons made with the 95th percentile of HBM study of the Irish adult population [57] represented by green dots and the 95th percentile of a German children population from the GerES study [31], represented by a blue dash.

**Figure 2:**
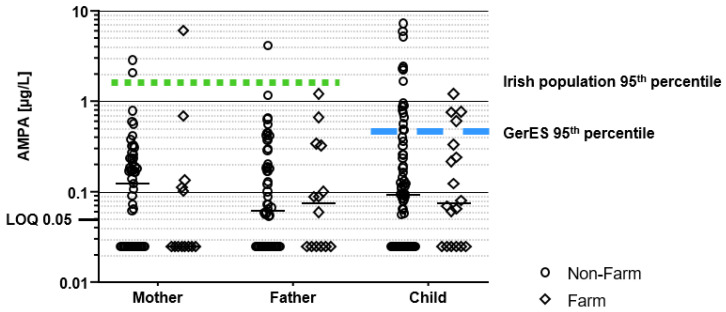
Urinary concentrations of AMPA by the family member (e.g., mother, father, child) and the family type (e.g., farm and non-farm. Comparisons made with the 95th percentile of HBM study of the Irish adult population [57] represented by green dots and the 95th percentile of a German children population from the GerES study [31], represented by a blue dash.

A graphical presentation of all individual concentrations is provided in Figure 1 for glyphosate and Figure 2 for AMPA and they are compared to a study of Irish adults [57] and a study of German children [31]

The chi-squared tests of independence showed no statistically significant differences in the frequency of quantifications when comparing all farm family members with non-farm family members. Of note, the farm fathers (the users of glyphosate the previous day) did not show statistically increased detections of glyphosate (or AMPA) compared to other fathers. Thus, we combined the two subsets (farm and non-farm families) and compared concentrations of children (*n* = 93) with fathers (*n* = 68) and mothers (*n* = 66). The combined dataset is presented in Supplementary Materials Table S2.

Children had the highest frequency of quantification for glyphosate (32%) and AMPA (61%); however, their concentrations were not significantly different from the fathers (25% and 59%) or mothers (18% and 56%), respectively. Therefore, to investigate potential relationships between different family members, we investigated correlations between the father’s concentrations and those of the mothers and children in their respective families. Only for farm family glyphosate concentrations was there a statistically significant, moderate correlation between the father’s concentration and that of the respective family members (R^2^ = 0.38; *p* < 0.05).

Although no statistically significant differences were found between farm and non-farm families regarding detection frequencies, the five-highest glyphosate concentrations among the farm families were contributed by the male farmers (that used glyphosate the day before). To further investigate this, the 14 pesticide users (i.e., farm fathers) were compared to the 53 non-farm fathers and the whole adult population (i.e., mothers and non-farm fathers) by the Mann–Whitney test. The test found statistically significant differences between the father types (e.g., farm vs. non-farm) and between the pesticide user and other adults (e.g., farm father vs. mothers and non-farm fathers). The geometric means and 95th percentile confidence intervals of the fathers’ groups did not significantly overlap (i.e., GM of 0.032 µg/L (0.025–0.037) vs. 0.106 µg/L (0.036–0.316)). However, these findings should be interpreted with caution due to the low sample size, the high number of left censored data and the correlation of concentrations for farm families.

### 3.3. Risk Assessment

As per Equation (1), the maximum urinary glyphosate and AMPA concentrations found among the adult participants corresponded to 2% and 0.2% of the EFSA Acceptable Daily Intake (ADI) of 0.5 mg/kg bw/day [1] guidance value. For the children, the highest concentration of glyphosate was from a non-farm child with a weight of 19 kg, which corresponded to 3% of the ADI. For AMPA, the highest value was also a non-farm child, with a weight of 18.6 kg, corresponding to 0.4 % of the ADI.

## 4. Discussion

The study investigated glyphosate and AMPA exposures among farm families and non-farm families and, thus, the potential for elevated baseline levels among families living with a pesticide user (e.g., residential and take-home exposures) or in a potentially glyphosate/AMPA-contaminated environment.

Although only farm families who reported using glyphosate on the day before sampling were included in the study, this did not result in a statistically significant impact on urinary glyphosate concentrations of the users or their families, when compared to the concentrations detected among non-farm families with no reported use prior to sampling. However, the farm fathers did have the highest frequency of glyphosate quantifications (43%) and the highest maximum glyphosate concentration (3.21 µg/L) compared to the farm family mothers (23%; max 0.23 µg/L) and children (17%; 0.23 µg/L). Further, the five-highest glyphosate concentrations were found within this group. However, concentrations in the farm fathers were lower than those reported in previous occupational studies [20,36,45], including Irish occupational studies (by the lead author) [34,35]. It is important to note that the urine samples of the farm fathers were collected the morning after spraying, as glyphosate has a very short half-life [58]. Urinary glyphosate concentrations are known to peak 1–3 h after task completion and rapidly decline thereafter [34]. However, the aim of this study was not to quantitatively capture the user’s exposure but to assess the whole farm family consistently, with each family member giving the urine sample the morning after spraying, in line with our aim to assess potential residential or take-home exposures.

Residential (e.g., living on a farm) or take-home exposures (e.g., living with an occupational user), possibly due to the worker bringing pesticide residues into the home on their work clothes, skin and personal items (e.g., mobile phones), had been identified in other studies [43,59,60,61,62] as a potential exposure source. We only found a moderate relationship between the farm father’s (i.e., pesticide sprayer) glyphosate concentrations and the respective family members, but exposure levels did not statistically differ between farm and non-farm families. Curwin and Hein [45] conducted a similar study in the US and found no statistical differences among the adult family members and the children of non-farm families had marginally higher levels of glyphosate than the farm children. However, recent studies have identified the need to further investigate exposure to subpopulations (e.g., families living close to agricultural fields) [63].

Non-farm family members were evaluated for environmental glyphosate and AMPA exposure concentrations measured in this study are comparable to concentrations reported in previous studies [20]. Although this is the first European human biomonitoring study to investigate all family members’ exposures to glyphosate, some studies investigated adults’ and children’s/adolescents’ exposures separately. Some of the most extensive HBM studies evaluating glyphosate exposures have been conducted in Germany. One HBM study among the adult population collected 399 urine samples over 15 years and found quantifiable glyphosate and AMPA in 32% and 40% of the samples, respectively. The highest median and maximum levels found over the years of the study were 0.18 µg/L and 2.8 µg/L, and 0.18 µg/L and 1.88 µg/L for glyphosate and AMPA, respectively [30]. A more recent study from Germany analysed 2144 urine samples from children and adolescents collected from 2015 to 2017 and found quantifiable levels in 52% and 46% of samples for glyphosate and AMPA, respectively. For glyphosate, the median and maximum levels were 0.10 µg/L and 1.11 µg/L, while for AMPA, the median was below the limit of quantification and the maximum level was 13.4 µg/L [31].

The HBM4EU initiative reviewed HBM-aligned studies for adults [64] and children [63]. For both the adult and children HBM4EU studies, results showed that glyphosate and AMPA exposure is widespread in the EU, with similar concentrations found in the current study. Overall, the concentrations in our study were in the same range as most EU studies. Only for AMPA did we find that the children in the current study had slightly elevated concentrations in the upper range, with 22.2% of the Irish farm children and 20% of the Irish non-farm children exceeding the 95th percentile of the German children (Figure 2). Similar to the other studies, the frequency of quantifiable glyphosate and AMPA among children and adolescents is observed as higher than in adults [31,63,64]. However, whether this results from physiological differences, such that children have higher ingestion of food and drink per kilogram bodyweight, increased exposure to the chemical from human behaviour (e.g., children playing outdoors with potential for pesticide exposure in the outdoor environment) or differing diets is uncertain.

There has been only one previous Irish HBM study investigating environmental glyphosate exposures, which found that 10 of the 50 samples collected in 2017 were quantifiable for glyphosate [38]. However, these results are not directly comparable to our study as they were analysed using a less sensitive analytical method (LOQ 0.5 µg/L). Recently, these samples were reanalysed with a more sensitive method (LOQ = 0.1 µg/L) and the frequency of quantifiable glyphosate increased to 76%, with a median, 95th percentile and maximum value of 0.23 µg/L, 0.89 µg/L and 1.52 µg/L, respectively. For AMPA, 72% of samples had quantifiable AMPA concentrations and the median, 95th percentile, and maximum values were 0.15 µg/L, 1.75 µg/L, and 2.25 µg/L, respectively [65]. Therefore, compared to our current study, glyphosate was more frequently quantifiable in samples collected in 2017 and had slightly higher concentrations. The marked decrease in detection frequency and reported concentration could reflect an increased awareness of glyphosate exposures since the IARC’s ‘Group 2A—probably carcinogenic to humans’ classification of glyphosate and also be due to increased national efforts to reduce pesticide consumption within the country (e.g., initiatives to increased plant biodiversity and sustainability, such as the European Green Deal [66] and Farm to Fork strategy [67]).

An interesting study finding was the higher frequency of quantification and concentration levels for AMPA across all the subgroups compared to the parent compound glyphosate. AMPA is an environmental breakdown product of glyphosate, with only minuscule amounts (<1%) of glyphosate metabolised to AMPA by humans [56]. Thus, the major share of AMPA excreted in urine most likely stems from direct, concurrent uptake of AMPA and not glyphosate. AMPA is a known environmental degradation product of glyphosate. Residues on foods, plants or soil can also leach into watercourses, directly exposing the general population to AMPA via ingesting contaminated food and water. It is important to evaluate AMPA exposures alongside glyphosate exposures [20], especially as AMPA has a similar toxicological profile to glyphosate [7,68]. Though the main source of AMPA is from the environmental breakdown of glyphosate, it has also been identified as a breakdown product of amino-polyphosphonate degradation fire retardants, anticorrosives and anti-scaling agents [69], as well as industrial detergents and cleaning products [70], which may contribute to overall exposure levels. Another explanation for the differences in the results for glyphosate and AMPA is the fast degradation of glyphosate in the environment, while AMPA persists longer in the environment [71]. A better understanding of the sources of AMPA in the environment and the possible intake routes (e.g., De Troeyer, Casas [72] observed associations with the proportion of agricultural land use around the residence) would assist in informing risk assessments and management [63].

To assess the risk, the worst-case exposure scenario (i.e., using maximum concentrations) was evaluated, although assessing maximum concentrations tends to overestimate the general averages for most analytes [73]. The maximum urinary glyphosate and AMPA concentrations corresponded to 3% or less of the Acceptable Daily Intake (ADI) for adults and children for both glyphosate and AMPA. Previous studies have used this calculation method [54], using the assumption that the daily intake of oral glyphosate excreted as unchanged glyphosate is approximately 20% (also applying to AMPA), based on animal studies, while more recent human volunteer studies have estimated the urinary excretion fraction to be closer to 1% for glyphosate [56,74], resulting in a 20-fold difference in intakes than in previous assessments. Therefore, to ensure the robustness of our evaluations, we used these lower, more conservative urinary fractions in this study’s calculations. However, this ADI-based assessment might only be valid under the assumption that glyphosate is not carcinogenic and has a threshold for effects. As long as no consensus on potential carcinogenic effects has been reached in the worldwide scientific community, including IARC, this interpretation should, therefore, be put under the caveat that the discussion is not finally concluded. Future outcomes on carcinogenicity would influence risk conclusions presented in the current study, which has an issue that has also been identified in other glyphosate studies [63,64]. Furthermore, there is an absence of an ADI for combined glyphosate and AMPA exposure (e.g., gly + AMPA), which has been previously suggested [75].

This study was the first in Europe to evaluate all family members, as well as to evaluate farm versus non-farm families, although there were some shortcomings. First, a more elaborate statistical analysis could not be performed due to the majority of glyphosate/AMPA samples below the LOQ, despite using one of the most sensitive methods so far (LOQ = 0.05 µg/L). As the study relied on volunteers, the study may have had a bias, as participants’ motives may have been due to their concern over pesticide use and a large proportion of families that participated indicated that they included organic foodstuffs within their weekly diet diary. This could affect the representativeness of the dataset when extrapolating information to the general public. Finally, the sampling campaign was halted during one of the main spraying seasons; thus, the exposure levels may not reflect environmental exposures during a spraying season with high pesticide use. The campaign was then ongoing during the COVID-19 pandemic, with adapted sampling protocols, heightened difficulty for recruitment, significant delays and only a small sub-group of farm families.

A major strength of the study was that the sampling protocols and documentation were developed to align with the HBM4EU initiative. The analytical method developed for the IMAGE project was selective and sensitive enough to detect both glyphosate and AMPA at environmental exposure levels and successfully participated in the inter-laboratory comparison study [50] conducted by the HBM4EU initiative [76]. This external quality assurance/quality control assessment ensures the standard of the method and enhances the comparability of these results with future studies utilising similar methods [63,64].

## 5. Conclusions

Assessing the potential glyphosate exposures among the general population and potentially exposed sub-populations is an important public-health measure, especially after the IARC classification of glyphosate as ‘probably carcinogenic to humans’. Our results can contribute to furthering the understanding of whole family exposures and information necessary for chemical regulatory and policy input. Thus, these results are particularly timely against the background of the pending re-evaluation of glyphosate in 2022 by the European Commission [77]. Moreover, our results advocate a combined assessment of glyphosate and AMPA exposures and document the need for further elucidating the relevance of direct AMPA intake and potential sources.

## Figures and Tables

**Table 1:** Participant information: personal and demographic characteristics. Data are presented as a number of observations or mean values (range) for parameters on a continuous scale.

Family Type	Participants	Sample Size (No)	Age in Years AM (Range)	BMI [kg/m^2^] AM (Range)
Non-farm	Adult (Male)	54	45 (29–57)	26.5 (19.5–36.2)
	Adult (Female) ^1^	53	43 (26–54)	23.9 (17.9–38.8)
	Child (Male)	37	11 (3–19)	18.4 (13.7–32.6)
	Child (Female) ^2^	37	10 (3–18)	17.5 (12.4–25.1)
Farm	Adult (Male) *	14	48 (39–60)	26.5 (21.1–34)
	Adult (Female) ^1^	13	43 (36–55)	25.5 (16.3–32.4)
	Child (Male)	14	10 (3–17)	17 (14.1–24.1)
	Child (Female)	4	9 (7–11)	18.6 (14.8–22)

Sample size (no); the number of samples within this subgroup, age (years), AM (range); the age of the participants given by arithmetic mean and the range (min–max) within this subgroup, BMI (kg/m^2^) AM (range); the Body Mass Index given as kilograms per meter of height squared given by arithmetic mean and the range (min-max) within this subgroup. * All males (no females) from the farm used glyphosate products the day before sampling. ^1^ One mother from the non-farm family and one from the farm family opted out of the study. ^2^ One child from the non-farm family gave two samples. The child missed the first-morning void and gave another sample on a different day.

**Table 2:** Human biomonitoring. Biological monitoring results (µg/L) grouped as family type (farm/non-farm) and participant (father/mother/child), with the number of families and family members, and describing the number of urinary samples, the percentage of quantifiable samples, range, median and P95.

	Family Type	Number of		Urine Levels (µg/L)				
		Families	Family Members	No.	% ≥ LOQ	Range	Median	P95
**Glyphosate**								
Father	Non-farm	54	54	54	20%	<LOQ–0.17	<LOQ	0.11
	Farm *	14	14	14	43%	<LOQ–3.21	<LOQ	2.49
Mother	Non-farm ^1^	54	53	53	17%	<LOQ–0.17	<LOQ	0.12
	Farm ^1^	14	13	13	23%	<LOQ–0.23	<LOQ	0.16
Children	Non-farm ^2^	54	74	75	36%	<LOQ–2.48	<LOQ	0.27
	Farm	14	18	18	17%	<LOQ–0.23	<LOQ	0.17
**AMPA**								
Father	Non-farm	54	54	54	59%	<LOQ–4.12	0.06	0.64
	Farm *	14	14	14	57%	<LOQ–1.22	0.07	0.86
Mother	Non-farm ^1^	54	53	53	60%	<LOQ–2.86	0.12	0.67
	Farm ^1^	14	13	13	38%	<LOQ–6.01	<LOQ	2.82
Children	Non-farm ^2^	54	74	75	60%	<LOQ–7.24	0.09	2.33
	Farm	14	18	18	67%	<LOQ–1.22	0.08	0.83

No.: the number of samples analysed within this subgroup; % ≥ LOQ: Percentage of samples above the limit of quantification; Range: Minimum to Maximum concentrations of glyphosate quantified in this subgroup. * All males (no females) from the farm used glyphosate products the day before sampling. ^1^ One mother from the non-farm family and one from the farm family opted out of the study. ^2^ One child from the non-farm family gave two samples. The child missed the first-morning void and gave another sample on a different day.

## Supplementary Materials

The following supporting information can be downloaded at: https://www.mdpi.com/article/10.3390/toxics10110690/s1, Supplementary Information: COVID-19 sampling protocols, Table S1: Creatinine-adjusted values; Table S2: Combined human biomonitoring results by family member.
