# Supplementary material for: A Human Biomonitoring Study Assessing Glyphosate and Aminomethylphosphonic Acid (AMPA) Exposures among Farm and Non-Farm Families
Source: Toxics. 2022 Nov 15;10(11):690. doi: 10.3390/toxics10110690 (PMC9697524; doi:10.3390/toxics10110690)
Supplement: Supplementary file 1 [file toxics-10-00690-s001.zip › toxics-2000020-supplementary.pdf]

toxics

MDPI

Supplementary Materials: A Human Biomonitoring Study Assessing Glyphosate and Aminomethylphosphonic Acid (AMPA) Exposures among Farm and Non-farm Families

Alison Connolly, Holger M. Koch, Daniel Bury, Stephan Koslitz, Marike Kolossa-Gehring, André Conrad, Aline Murawski, James A. McGrath, Michelle Leahy, Thomas Brüning and Marie A. Coggins [1]

COVID-19 sampling protocols

The IMAGE project sampling protocols were developed prior to the COVID-19 pandemic, and the campaign commenced in autumn of 2019. On the 29th of March 2020, within the Republic of Ireland governmental restrictions were issued relating to travel and movement in Ireland due to the COVID-19 pandemic, which included staying at home except for essential travel and social distancing from other people (https://www.gov.ie/en/publication/6973bc-daily-briefing-on-the-governments-response-to-covid-19-monday-30-mar/).

Before the re-commencement of the sampling campaign, sampling protocols had to be adapted to comply with public health guidelines and to ensure the safety of researchers and participants. Risk assessments were updated and included extra controls. The participating families were asked to sign an updated consent form which included information on the new sampling strategy and asking confirmation from participants that they would not participate in the study if they were confirmed with COVID-19, had symptoms or were on quarantine as suspected to have the virus. Additionally, samples were no longer collected from participants that were cocooning (i.e. the extremely medically vulnerable staying at home for their safety) and it was ensured that there was a minimum of 2 meter distance between participants and the researcher at all times and that the researcher would not enter the participant's home during sample collection.

For fieldwork, participants were asked to collect urine samples in the provided containers which were to be placed in sampling bags and to freeze until collection. The collection of urine samples was to be conducted outside of the home while maintaining a minimum of 2 meter distance between participants and the researcher at all times. The researcher used disposable nitrile gloves and facemasks during the collection and all collected items were wiped with antiviral wipes (70% Isopropanol alcohol). Samples were transferred within a plastic box with clip-down lids that contained superabsorbent absorbent liners with (1.2 l capacity). All paperwork was left unopen for a minimum of one week, to reduce the potential of virus transfer.

When aliquoting the samples for shipment, the researcher wore disposable gloves, goggles, disposable face mask and a disposable lab coat and handled the samples within a class 2 biosafety cabinet which was disinfectant before and after use. The excess urine samples were disposed of as samples with a potential to contain COVID-19 virus via competent bio-waste disposal company.

Supplementary Table S1: Creatinine adjusted values

Biological monitoring results given as creatinine adjusted values (ug/g creatinine) grouped as family type (farm/non-farm) and participant (e.g. father/mother/child), with the number of families and family members, and describing the number of urinary samples, the percentage of quantifiable samples, range, median and P95.

	Family type	Number of		Urine Levels (µg/g creatinine)				
		Families	Family Members	No.	% ≥ LOQ	Range	Median	P95
**Glyphosate**								
**Father***	**Non-farm**	54	54	54	20%	0.01 - 0.27	0.02	0.1
	**Farm**	14	14	14	43%	0.01 - 3.74	0.02	2.2
**Mother[1]**	**Non-farm**	54	53	53	17%	0.01 - 0.15	0.03	0.1
	**Farm**	14	13	13	23%	0.01 - 0.16	0.04	0.1
**Children[1]**	**Non-farm**	54	74	75	36%	0.01 - 3.77	0.03	0.3
	**Farm**	14	18	18	17%	0.01 - 0.29	0.04	0.2
**AMPA**								
**Father***	**Non-farm**	54	54	54	59%	0.01 - 2.75	0.05	0.4
	**Farm**	14	14	14	57%	0.02 - 1.18	0.06	0.7
**Mother[1]**	**Non-farm**	54	53	53	60%	0.01 - 3.33	0.08	0.7
	**Farm**	14	13	13	38%	0.01 - 3.5	0.04	1.8
**Children[1]**	**Non-farm**	54	74	75	60%	0.01 - 11.95	0.08	2.6
	**Farm**	14	18	18	67%	0.02 - 1.52	0.12	1.5

No.: the number of samples analysed within this subgroup; % ≥ LOQ: Percentage of samples above the limit of quantification; Range: Minimum to Maximum concentrations of glyphosate quantified in this subgroup; the median and the 95th percentile. *All males (no females) from the farm used glyphosate products the day before sampling 1. One mother from the non-farm family and one from the farm family opted out of the study.

Supplementary Information Table S2: Combined human biomonitoring results by family member

Biological monitoring results (μg/L) grouped by family member (e.g. father/mother/child) combining both family types (farm and non-farm), with the number of families and family members, and describing the number of urinary samples, the percentage of quantifiable samples, range, median and P95.

	Number of		Urine Levels (μg/L)				
	**Families**	**Family Members**	**No.**	**% ≥ LOQ**	**Median**	**P95**	**Max**
**Glyphosate**							
**Father***	68	68	68	25	< LOQ	0.51	3.21
**Mother[1]**	68	66	66	18	< LOQ	0.21	0.23
**Children[2]**	68	92	93	32	< LOQ	0.26	2.48
**AMPA**							
**Father***	68	68	68	59	0.06	0.66	4.12
**Mother[1]**	68	66	66	56	0.01	0.76	6.01
**Children[2]**	68	92	93	61	0.08	2.26	7.24

No.: the number of samples analysed within this subgroup; % ≥ LOQ: Percentage of samples above the limit of quantification; the median values, the 95th percentile and the maximum values found for each family member. *All males (no females) from the farm used glyphosate products the day before sampling. 1. One mother from the non-farm family and one from the farm family opted out of the study. 2. One child from the non-farm family gave two samples. The child missed the first morning void and gave another sample on a different day.
